# Clinical Presentation and MRI Characteristics of Appendicular Soft Tissue Lymphoma: A Systematic Review

**DOI:** 10.3390/diagnostics12071623

**Published:** 2022-07-04

**Authors:** Sebastian Weiss, Valentin Weisse, Alexander Korthaus, Peter Bannas, Karl-Heinz Frosch, Carsten Schlickewei, Alexej Barg, Matthias Priemel

**Affiliations:** 1 Department of Trauma and Orthopaedic Surgery, University Medical Center Hamburg-Eppendorf, 20251 Hamburg, Germany; vweisse@me.com (V.W.); a.korthaus@uke.de (A.K.); k.frosch@uke.de (K.-H.F.); c.schlickewei@uke.de (C.S.); al.barg@uke.de (A.B.); priemel@uke.de (M.P.); 2 Department of Diagnostic and Interventional Radiology and Nuclear Medicine, University Medical Center Hamburg-Eppendorf, 20251 Hamburg, Germany; p.bannas@uke.de; 3 Department of Trauma Surgery, Orthopaedics and Sports Traumatology, BG Klinikum Hamburg, 21033 Hamburg, Germany; 4 Department of Orthopaedics, University of Utah, Salt Lake City, UT 84108, USA

**Keywords:** soft tissue lymphoma, MRI, soft tissue masses, extremities, radiology, MSK

## Abstract

Appendicular soft tissue lymphoma (ASTL) is rare and is frequently misinterpreted as soft tissue sarcoma (STS). Studies investigating magnet resonance imaging (MRI) characteristics of ASTL are scarce and showed heterogenous investigation criteria and results. The purpose of this study was to systematically review clinical presentations and MRI characteristics of ASTL as described in the current literature. For that purpose, we performed a systematic literature review in compliance with the Preferred Reporting Items for Systematic Reviews and Meta-Analyses (PRISMA) guidelines. Patient demographics, clinical presentation and MRI imaging characteristics of ASTL were investigated, resulting in a total of nine included studies reporting a total of 77 patients. Signal intensity of lymphoma compared to muscle tissue was mostly described as isointense (53%) or slightly hyperintense (39%) in T1-weighted images and always as hyperintense in proton-and T2-weighted images. Multicompartmental involvement was reported in 59% of cases and subcutaneous stranding in 74%. Long segmental involvement was present in 80% of investigated cases. Involvement of neurovascular structures was reported in 41% of cases and the presence of traversing vessels in 83% of patients. The presence of these findings should lead to the inclusion of ASTL in the differential diagnosis of soft tissue masses.

## 1. Introduction

Primary extranodal lymphoma is commonly known to manifest in a variety of tissues, such as the gastrointestinal tract [1], central nervous system [2], skin [3], bone [4] and the respiratory system [5]; however, primary appendicular soft tissue manifestation of lymphoma (ASTL) is rare. Merely eight out of 7000 patients (0.1% of patients) with malignant lymphoma have been reported to present with soft tissue manifestation of the extremities [6]. Another study found 472 soft tissue lymphoma among 38,484 (1.2% of patients) reviewed cases of soft tissue tumors [7]. Most commonly, involvement of muscle in lymphoma manifestations is due to secondary infiltration from hematogenous spread [8], adjacent bone [9] or lymph nodes [10]. Primary lymphoma manifestation in skeletal muscle and other soft tissues is rare [11,12].

It is supposed that primary intramuscular manifestation of lymphoma may be derived from deviant lymph nodes, which might not be histologically recognizable at the time of diagnosis [6]. MRI is widely considered to be the imaging modality of choice to detect and differentiate between soft tissue masses [13,14,15]. However existing studies investigating MRI characteristics of ASTL are small scaled or consist of case series and case reports [16]. Moreover, investigation criteria among the existing studies are vastly heterogenous and differ in results. Consequently, ASTL are frequently misdiagnosed or not considered in the differential diagnosis of appendicular soft tissue tumor presentations [17]. The most frequently suspected differential diagnosis in ASTL is soft tissue sarcoma (STS) [18,19]. STS usually receives radical excisional surgery, whereas ASTL usually responds well to chemotherapy with or without additional radiation therapy [20]. A misdiagnosis may subsequently result in inadequate surgical or oncologic therapy and could put patients at risk for compromised function or even loss of a limb. Moreover, wrongful major excisional therapy deprives the physician of a useful clinical parameter to measure treatment outcome as it would be provided by an in-situ tumor [20]. Additionally, a correct radiological differential diagnosis may assist the pathologist in performing adapted immunohistological stains [21]. The purpose of this study was therefore to systematically review clinical presentations and MRI characteristics of ASTL as described in the current literature and to subsequently present the currently heterogenous and scarce data in a clearly structured and plain manner. This may offer guidance for the radiologist as well as the clinician in suspecting the correct differential diagnosis in soft tissue tumor presentations.

## 2. Materials and Methods

This systematic review was performed and reported in compliance with the Preferred Reporting Items for Systematic Reviews and Meta-Analyses (PRISMA) guidelines [22]. Moreover, this study has been registered at the International Prospective Register of Systematic Reviews (PROSPERO) (ID: CRD42022310322). All analyses conducted in this systematic review were performed by two independent investigators. According to protocol, discrepancies were resolved by discussion and consensus.

### 2.1. Literature Search

Medline and Scopus databases were searched for original studies that reported MRI characteristics of ASTL by using the following search term: ((Soft Tissue Lymphoma) OR (Subcutaneous Lymphoma) OR (Lymphoma Extremities) OR (Lymphoma Appendicular) OR (Intramuscular Lymphoma) OR (Lymphoma Muscle) OR (Limb Lymphoma) OR (Musculoskeletal Lymphoma)) AND ((MRI) OR (Magnetic Resonance Imaging)). References of included articles were screened for potentially missed articles. We continuously updated the search until 1 March 2022. Peer reviewed journal articles which investigated MRI features of ASTL were included. In studies using a multimodal imaging approach, only data of patients who underwent MRI were included. Exclusion criteria were studies which did not sufficiently describe MRI characteristics, case series with less than three patients or studies conducted before 1990. Furthermore, review articles, case reports, non-peer-reviewed articles, studies that investigated solely lymphoma manifestation of skin, subcutaneous tissue or primary osseus origin and non-English language studies were excluded. Immunocompromised patients with conditions such as acquired immunodeficiency syndrome (AIDS) or relapse after chemotherapy were excluded due to evidence for possible associated atypical imaging features of lymphoma [23,24,25]. In studies with multifocal localization of soft tissue lymphoma, patients without appendicular localization of soft tissue lymphoma were excluded. Multifocal manifestation of disease was included.

### 2.2. Quality Assessment of Included Studies

The critical appraisal tool to assess the quality of cross-sectional studies (AXIS) was used to determine the methodological quality and risk of bias of the included studies [26]. The AXIS tool evaluates study design and reporting quality as well as the risk of bias in analytical cross-sectional studies and contains 20 items [27]. As previously shown to be effective [28,29], the tool was modified to match the given study population. The remaining 12 questions (Table 1) were utilized to evaluate the risk of bias of the included studies and could be answered with “yes” or “no”. Answer “yes” was assigned to a score of 1, answers “no” or “not reported” (questions 11 and 12) were assigned to a score of 0, resulting in a maximum score of 12. 

In accordance with Menolotto et al. [29], a score ranging from 10–12 was assigned to a low risk of bias and high-quality study, a score ranging from 7 to 9 was considered to represent a medium risk of bias and medium-quality study and a score of 6 or lower represented a high risk of bias and a low-quality study. Separately, we evaluated if the investigation has been conducted by more than one researcher to ensure interrater reliability.

### 2.3. Data Extraction

Universal study characteristics such as year of publication, number of included patients and utilized imaging modalities were extracted. Moreover, we extracted clinical and demographic information, including age, sex, localization of lesion, histopathological diagnosis, whether there has been mono- or multifocal involvement, presence of lymphadenopathy, indication of pain or discomfort, presence of muscle enlargement, the average duration of symptoms until presentation, LDH levels and presence of B symptoms. Subsequently, MR imaging characteristics of ASTL were extracted. Investigations included lesion size, signal intensity in T1-, T2-, and proton-density (PD)- weighted images as well as fat-suppressed (FS) (STIR) images compared to unaffected muscle and fat tissue, contrast enhancement pattern of the tumor and its adjacent fascial planes, demarcation, number of affected muscles, presence of multicompartmental involvement, differentiation of diffuse or focal soft tissue involvement, subcutaneous stranding and skin thickening, long segmental involvement (growth of tumor oriented along of muscle fascicles) [30], involvement of neurovascular structures and bone, traversing of vessels and the presence of edema, tumor encapsulation and necrosis. Moreover, we extracted information concerning diffusion weighted imaging (DWI). Contrast enhancement data were grouped in heterogenous patterns (including thick bandlike and marginal septal enhancement) and homogeneous enhancement patterns. Descriptions of demarcation patterns were grouped into well-defined vs. poorly-defined.

### 2.4. Data Synthesis and Statistical Analysis

Collected data were evaluated using means of descriptive statistics (absolute and relative frequencies). In instance of missing information in included patients, descriptive statistics were calculated excluding the affected patients of the respective variable. Standard deviations were calculated when feasible. Statistical analyses were performed using IBM^®^ SSPS^®^ Statistics, v.27 (IBM, Armonk, NY, USA).

## 3. Results

### 3.1. Literature Search

**Figure 1:**
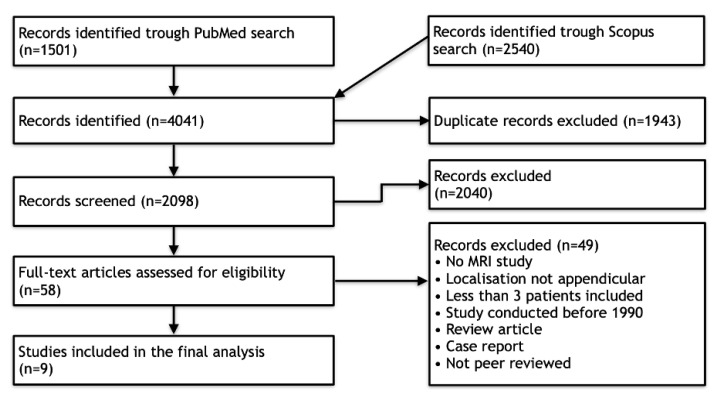
Preferred Reporting Items for Systematic Reviews and Meta-Analyses (PRISMA) flowchart.

The initial screening resulted in 1501 articles identified on PubMed Medline and 2540 articles identified on the Scopus database. After duplicate removal (n = 1943), titles and abstracts of 2098 articles were screened, leading to 2040 excluded articles. The remaining 58 full text articles were assessed for eligibility, which led to further exclusion of 49 articles. Ultimately, nine articles were included in the final analysis (Figure 1).

### 3.2. Quality Assessment

Nine studies were assessed for risk of bias and reporting quality according to our modified AXIS tool (Table 2). Three studies showed low risk, four studies showed medium risk and two studies showed high risk of bias. Detailed AXIS scores for individual included studies can be found in Table 3. Moreover, only three studies reported more than one investigator [18,19,30].

### 3.3. Data Extraction

This systematic review included nine studies with a total number of 77 patients. All included studies were of a retrospective nature. Study parameters, patient demographics and clinical data are displayed in Table 3. Seven of the nine studies included small sample sizes ranging from two to eight patients, only studies by Chun et al. (n = 20) and Suresh et al. (n = 24) included more than ten patients [18,30]. Five Studies utilized MRI as the primary imaging modality and four studies investigated ASTL characteristics in several imaging modalities, including MRI. Due to high variation of evaluated parameters in the included studies, the total number of patients for statistical analysis of each parameter shows high fluctuation. Imaging protocols were varying, using 0.5 to 3 Tesla systems. Sex, age and localization of lymphoma could be identified in 72 of 77 patients (94%). Of these 72 patients, 40 were male (56%) and 32 were female (44%). Average age was 59 ± 17 years (range 5–91). The most frequently reported location was the thigh (n = 20; 28%), followed by the upper arm (n = 9; 13%), calf (n = 7; 10%) and trunk (n = 7; 10%). The histopathological diagnosis could be identified in 69 cases in which diffuse large B-cell lymphoma represented the most common subtype, described in 28 (41%) cases. This was followed by not further differentiated descriptions of non-Hodgkin lymphoma in 11 (16%) cases, eight (10%) cases of follicular lymphoma and 23 (33%) cases of various other histological subtypes. Five of 33 identifiable patients (15%) showed multifocal involvement, whereas 28 patients (85%) showed unifocal manifestation. Lymphadenopathy was present in seven out of 31 (23%) cases in which lymphadenopathy was investigated. Pain or discomfort were reported in seven of 13 cases (54%), and swelling or enlargement, either clinical or radiologically (Table 3) in 37 of 38 cases (97%). The average duration of symptoms until presentation was 11 ± 8 weeks (range 4–24) but was only described in nine patients. None of the included studies investigated pretherapeutic LDH levels or B symptoms. Only one study described lesion sizes in 24 patients with a mean cranio-caudal length of 12.7 cm and the anterior-posterior and transversal diameters measuring 6 × 5.95 cm [18].

**Table 3:** Study parameters including demographics and clinical data (sorted by year).

Study Parameters				Demographics		Clinical Data		
Author/Year	AXIS Score	No. of Patients Included	Imaging Modalities	Sex	Age Range (Years)	Regional Lymphadenopathy (Yes/No)	Pain/Discomfort (Yes/No)	Swelling/Enlargement of Muscle (Based on Clinical vs. MRI Examination) (Yes/No)
Hosono et al., 1995 [31]	6	4	MRI/CT	2F 2M	58–65	N/A	2/4	4/4 (via MRI)
Beggs et al., 1996 [32]	5	4	MRI/ CT/ Ultrasound	2F 2M	42–68	1/4	3/4	3/4 (clinical)
Eustace et al., 1996 [33]	8	2	MRI	2F	67–68	N/A	1/2	2/2 (via MRI)
Lee et al., 1997 [34]	8	3	MRI/CT/ Radiography/ Scintigraphy	3F	15–80	N/A	1/3	3/3 (clinical)
Carroll et al., 2007 [19]	9	7	MRI	2F 5M	56–68	6/7	N/A	N/A
Suresh et al., 2008 [18]	10	24	MRI	10F 14M	35–91	N/A	N/A	N/A
Surov et al., 2010 [11]	11	5	MRI/CT	N/A	N/A	N/A	N/A	5/5 (via MRI)
Chun et al., 2010 [30]	10	20	MRI	6F 14M	5–90	0/20	N/A	20/20 (via MRI)
Surov et al., 2014 [35]	9	8	MRI	5F 3M	58–73	N/A	N/A	N/A
**Total**	**2 Low quality** **4 Medium quality** **3 High quality**	**77**	**4 MRI** **5 Multimodal**	**32F** **40M** **5 N/A**	**Range: 15–91** **Mean ± SD: 59 ± 17**	**7 Yes** **24 No** **46 N/A**	**7 Yes** **6 No** **64 N/A**	**37 Yes** **1 No** **39 N/A**

**Figure 2:**
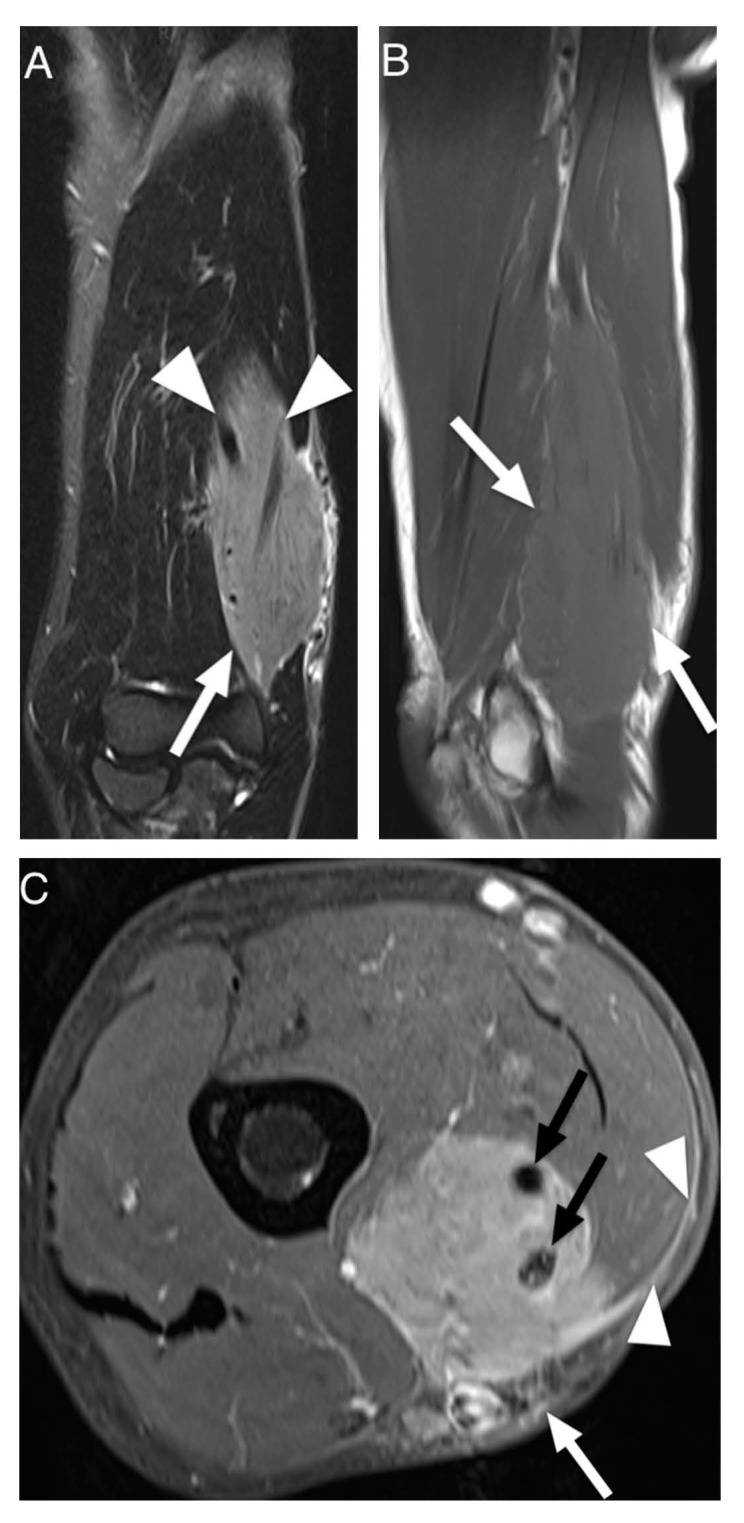
MRI of a 73-year-old man presenting with non-Hodgkin lymphoma manifestation in the flexor compartment of the upper arm. (**A**). Coronal T2-weighted TIRM image shows tumor of hyperintense signal intensity compared to muscle (arrow) and growth along the brachial neurovascular bundle (arrowheads). (**B**). Sagittal T1-weighted image shows long segmental involvement (arrows) and slightly hyperintense signal intensity of tumor in comparison to adjacent skeletal muscle. (**C**). Transversal contrast enhanced fat-saturated T1-weighted image shows homogenous contrast-enhancement of the lymphoma with encasement of the brachial neurovascular bundle (black arrows). Furthermore, subcutaneous stranding (white arrow) and contrast enhancement of the deep peripheral fascia (arrowheads) can be seen.

**Figure 3:**
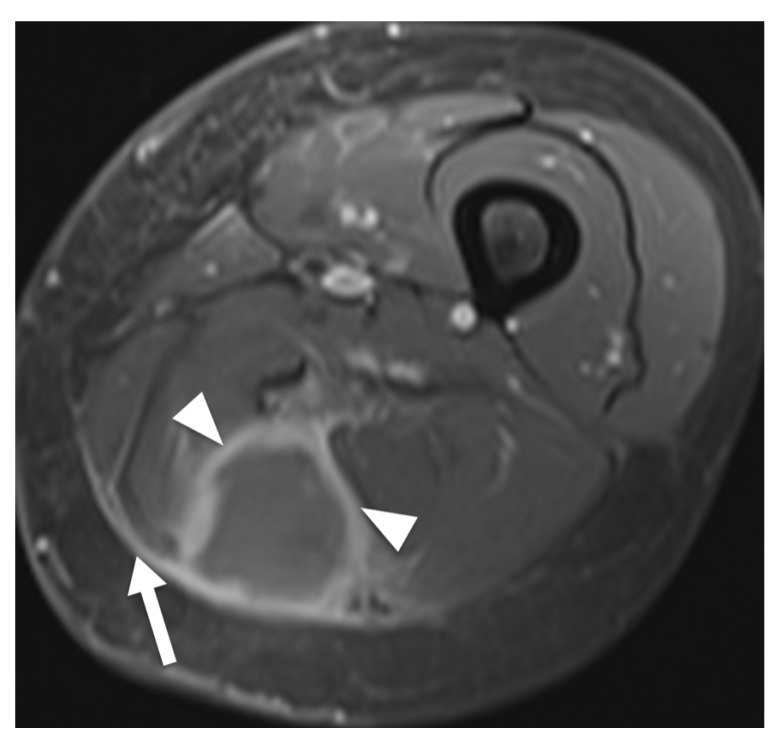
MRI of a 68-year-old woman presenting with non-Hodgkin lymphoma manifestation in the thigh. Transversal contrast enhanced fat-saturated T1-weighted image shows a tumor in the posterior compartment of the thigh, revealing predominant enhancement of tumor margins (arrowheads). Moreover, signal alteration of fascia lata can be noted (arrow).

Results of extracted MRI data are displayed in Table 4. Signal intensity of lymphoma compared to muscle tissue was mostly described as isointense (53%) or slightly hyperintense (39%) in T1W images and always as hyperintense in T2W images (Figure 2). The majority of T2W images revealed either isointense (47%) or hypointense signal intensity (45%) of lymphoma compared to fat. Hyperintense signal intensity compared to muscle was reported in all PD-weighted (n = 3) and STIR (n = 18) images. In fat-suppressed T2W images, one lesion (5%) showed isointense signal intensity to muscle, nine (43%) lesions showed isointense signal intensity to fat, three (14%) lesions showed intermediate signal intensity between fat and muscle and eight (38%) lesions showed hyperintense signal intensity compared to fat. None of the included articles investigated signal intensity in T1W, PD or STIR images compared to fat. Contrast enhanced images mainly showed homogenous enhancement (62%). One study differentiated further and described the presence of thick peripheral bandlike enhancement and marginal septal enhancement (Figure 3) [30]. Furthermore, the said series reported thick irregular enhancement of deep and superficial fascia in 16 patients (84%) (Figure 2 and Figure 3) whereas isolated enhancement of deep fascia was present in one patient (5%). Two (11%) patients did not show enhancement of fascia. Margins were described as poorly defined in 22 cases (61%) and as well-defined in 14 cases (39%). Multiple affected muscles were described in 19 of 30 (63%) identified cases. The presence of multicompartmental involvement was described in 36 of 61 (59%) of patients (maximum four compartments). Additionally, behavior of ASTL was reported as “often not confined to one muscle compartment” in one study [34]. Appearance in MRI was reported as a focal mass in 22 (59%) of cases and as diffuse abnormality of signal intensity in 15 cases (41%). Hereby, results were vastly heterogenous with several studies exclusively reporting diffuse abnormalities of signal intensity [11,33,34] while Carrol et al. reported a focal tumor mass in seven out of seven patients (100%) and Chun et al. reported focal manifestation in 15 out of 20 cases (75%) [19,30]. Subcutaneous stranding was examined in 34 cases and was present in 25 (74%) of these cases (Figure 2). Moreover, skin thickening was documented in 19 of 50 (38%) investigated patients. Long segmental involvement was reported in 20 of 25 (80%) of investigated cases (Figure 2). One study reported growth along neurovascular bundles with partial or complete encasement of these structures in seven of 24 cases (29%) [18]. Furthermore, Carroll et al. reported complete encasement of adjacent neurovascular structures in two cases and partial encasement in three of five cases not limited to the subcutaneous fat [19]. (Figure 2). Signal intensity abnormalities of bone marrow were described in nine of 49 (18%) examined patients, traversing vessels were found in 20 of 24 (83%) cases and peritumoral edema was noted in 15 of 31 (48%) cases. None of the included studies described signs of encapsulation or necrosis. Only one of the included studies provided DWI data and reported low signal intensity in apparent diffusion constant (ADC) images in all 10 investigated patients presenting with soft tissue lymphoma. Thereby, computed ADC values were ranging from 0.60–0.90 mm^2^ s^−1^ (mean: 0.76 ± 0.10; median: 0.78). Additionally, fusion images of DWI and HASTE, STIR or T2W images showed high signal intensity in all evaluated cases [35].

## 4. Discussion

**Figure 4:**
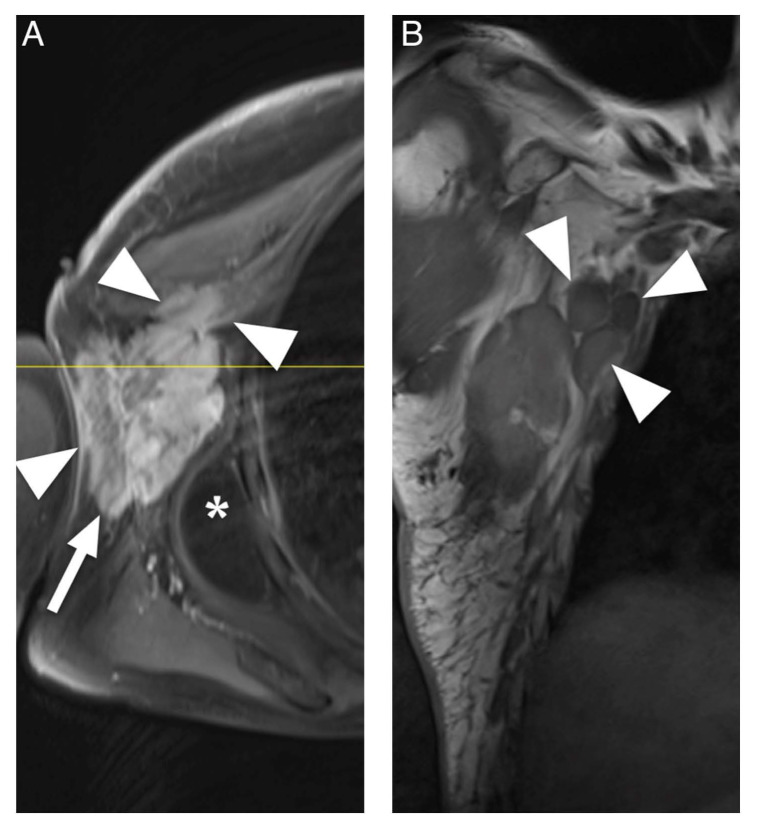
MRI of a 67-year-old man presenting with a predominantly subcutaneous manifestation of non-Hodgkin lymphoma in the right axilla. (**A**). Transversal contrast enhanced fat-saturated T1-weighted image shows a lobulated tumor (arrow) with inhomogeneous enhancement pattern which infiltrates into the pectoralis major and minor muscles and skin (arrowheads). Moreover, an accompanying lipoma can be noted (asterisk). (**B**). Coronal T1-weighted image shows associated lymphadenopathy (arrowheads).

The aim of this systematic review was to identify characteristic clinical and MRI features of appendicular soft tissue lymphoma as currently described in the literature. Diffuse large B-cell lymphoma was the most commonly reported histological subtype and is also the most common type of lymphoma [36]. Clinically, ASTL presents with swelling or enlargement of muscle, which may be explained by edema due to venous or lymphatic obstruction [34,37] or actual tumor growth [33]. Moreover, regional lymphadenopathy, which may be confluent, can be present [17], (Figure 4). Signal intensity in T1W sequences consistently showed iso- to slightly hyperintense signal intensity compared to skeletal muscle. T2W sequences comparing signal intensity to skeletal muscle frequently showed hyperintense signal intensity whereas reports of signal intensity compared to fat were widely inconsistent, describing hyperintense [34] and intermediate [30] but mostly hypointense signal intensity [11,18]. The results of Lee et al. might be explained by the use of spin echo sequences, which today are widely replaced by fast spin echo (FSE) sequences and result in lower signal intensity of fat than current FSE sequences [18,32].

Two studies investigated signal intensity in STIR sequences [18,32], reporting hyperintense signal intensity in all 18 cases and one study investigated signal intensity in PDW sequences reporting hyperintense signal intensity in all three patients [32].

In T2W fat-suppressed images, signal intensity was widely inconsistent. Therefore, we conclude that iso- to slightly hyperintense signal intensity in T1W sequences and hyperintense signal intensity in T2W, STIR and PDW images compared to muscle seem to be characteristic for ASTL. Homogenous contrast enhancement was reported more frequently than heterogenous enhancement and appears to be more characteristic. A previous study described enhancement of deep and superficial fascia, these findings might be related to the tendency of infiltrative, multicompartmental growth pattern of ASTL [30]. Margin descriptions of ASTL were heterogenous, ranging from poorly to well-defined. These findings are consistent with Gao et al. as ASTL margins in MR imaging appear to be nonspecific [16]. This heterogeneity might be attributed to differences in subjective MRI interpretation between studies. Multicompartmental involvement was defined as affected muscles of multiple fascial muscle compartments [11,18,30,31,32,33,34] or as involvement of various tissues [19] and was described in 36 of 61 (59%) patients (Figure 4). This feature can be useful to differentiate ASTL from soft tissue sarcoma, which are generally known to respect compartmental boundaries [15,21,34]. The presence of subcutaneous stranding was a commonly reported attribute of ASTL [13,18,30,32] and seems to be common in ASTL presentations. Explanations for these findings could be lymphomatous infiltration or reactive edema [19,38]. Furthermore, long segmental involvement, also called cone like involvement, of tumor seems to be a key feature of ASTL, being present in 20 of 25 included patients (80%), and can present inter- or intramuscularly [30,31,34]. Involvement of neurovascular structures was reported in 12 of 29 (41%) cases [18,19]. Furthermore, a previous study evaluating CT appearance of soft tissue lymphoma has found encasement of vascular structures in confluent lymphadenopathy associated with soft tissue lymphoma in six out of 13 patients (46%) [20]. These findings might be due to infiltration along lymphatic vessels accompanying the neurovascular bundle [18]. Signal intensity abnormalities of bone marrow were occasionally described and may present edema or lymphomatous infiltration [14,34,39]. They usually present without destruction of cortical bone [10] and might be due to spread of tumor through intracortical channels, first described by Hicks et al. in cases of primary osseous lymphoma [9,14]. Additionally, the presence of traversing vessels seems to be characteristic for ASTL as it was present in 20 of 24 (83%) investigated patients (Figure 4). Necrosis is generally considered as being absent in soft tissue lymphoma manifestations before treatment [8,14,37,40]; however, there are rare reports of necrosis being present in MRI of soft tissue lymphoma manifestations [18,41]. Diffusion weighted imaging seems to pose a further asset in the characterization of soft tissue lymphoma. Reported low ADC values coincide with high cellularity of lymphoma [35,42] and seem to be significantly lower than in other malignancies such as soft tissue sarcoma and lymph node metastasis [35,43,44]. This finding might be related to the tendency of soft tissue sarcoma and metastasis to express a more heterogenous, less dense cellularity than soft tissue lymphoma [44]. Moreover, DWI could be of use to monitor the treatment response, as it has been shown, that ADC values in soft tissue sarcoma increase after radiotherapy [45]. None of the included studies investigated presence of B symptoms; this may be due to reports of soft tissue lymphoma often not presenting with symptoms such as fever, weight loss and excessive night sweat [20,41]. Moreover, alteration of lactate dehydrogenase levels seems to be a possible clinical parameter of ASTL [46,47], nevertheless, none of the included studies evaluated this variable. 

## 5. Limitations

The small number of included studies and their patients is owed to the rarity of ASTL. Furthermore, the heterogeneity of investigated clinical and MRI characteristics, arose from varying methodology, interpretation and definition of features by investigators. The exclusively retrospective nature of the included studies might pose a risk for detection bias, as investigators are primed to look for certain variables. Furthermore, only three studies reported more than one investigator, this leads to suspected compromised interrater reliability in six of nine included studies and reduces their quality. Overall quality assessment reported a low risk of bias in only three of nine studies, leading to possibly compromised conclusions. Several studies did not investigate variables in a systematic, clearly structured manner leading to different variables described for each patient and in some cases uncertainty if the affected variable was merely not described or not present.

## 6. Conclusions

Detailed data in the current literature on the presentation of ASTL is scarce and lacks structure, yet some frequently present characteristics can be found. Clinically, ASTL shows an unspecific presentation with general swelling and possible association with pain and regional lymphadenopathy. The presence of hyperintensity in PDW, STIR and T2W MR sequences and iso- to slightly hyperintense signal intensity in T1W sequences, multicompartmental involvement, subcutaneous stranding, long segmental involvement, growth along neurovascular bundles with partial or complete encasement of these structures and the presence of traversing vessels should lead to the inclusion of ASTL in the differential diagnosis of soft tissue masses. The quality assessment and limitations of the included studies emphasize the need to interpret results with caution and highlight a demand for an appropriately sized and clearly structured approach in future research. 

## Figures and Tables

**Table 1:** Modified AXIS tool [26].

Question No.	Introduction
**Q1**	Were the aims/objectives of the study clear?
	**Methods**
**Q2**	Was the study design appropriate for the stated aim(s)?
**Q3**	Was the sample size justified?
**Q4**	Was the target/reference population clearly defined, did the sample frame represent the defined target/reference population and was the selection process appropriately?
**Q5**	Were the methods sufficiently described to enable them to be repeated?
	**Results**
**Q6**	Were the basic data adequately described?
**Q7**	Were the results internally consistent?
**Q8**	Were the results presented for all the analyses described in the methods?
	**Discussion**
**Q9**	Were the authors discussions and conclusions justified by the results?
**Q10**	Were the limitations of the study discussed?
	**Other**
**Q11**	Were there any funding sources or conflicts of interest that may affect the authors interpretation of the results?
**Q12**	Was ethical approval or consent of participants attained?

**Table 2:** Assessment of included studies for risk of bias and reporting quality, according to the modified AXIS tool [26].

AXIS Score	Risk of Bias	Reporting Quality	Number of Studies
0–6	High	Low	**2**
7–9	Medium	Medium	**4**
10–12	Low	High	**3**

**Table 4:** Summarized MRI characteristics of included studies (sorted by year).

Author/Year	T1W ^1^	T2W ^1^	T2W ^2^	PD ^1^	STIR ^1^	T1W Contrast Enhancement	Margins	No. of Affected Compartments	Long Segmental Involvement
Hosono et al., 1995 [31]	2 Slightly hyperintense 2 Isointense	3 Hyperintense 1 N/A	N/A	N/A	N/A	2 Homogeneous 2 N/A	N/A	1 Multiple 3 Single	2 Yes 2 N/A
Beggs et al., 1996 [32]	3 Slightly hyperintense 1 N/A	3 Hyperintense 1 N/A	N/A	3 Hyperintense 1 N/A	2 Hyperintense 2 N/A	2 Heterogeneous 2 N/A	1 Well-defined 3 poorly-defined	3 Multiple 1 Single	N/A
Eustace et al., 1996 [33]	2 Isointense	2 Hyperintense	N/A	N/A	N/A	2 Homogeneous	1 Well-defined 1 N/A	1 Multiple 1 Single	N/A
Lee et al., 1997 [34]	3 Isointense	N/A	3 Hyperintense	N/A	N/A	N/A	N/A	“Often more than one”	3 Yes
Carroll et al., 2007 [19]	5 Iso to slightly hyperintense 1 Isointense 1 Hyperintense	7 Hyperintense	N/A	N/A	N/A	5 Homogeneous 2 Heterogeneous	4 Well-defined 3 Poorly-Defined	5 Multiple 2 Single	N/A
Suresh et al., 2008 [18]	15 slightly hyperintense 8 Isointense 1 N/A	14 hyperintense 10 N/A	14 hypointense 10 N/A	N/A	16 Hyperintense 8 N/A	4 Homogeneous 9 Heterogeneous 11 N/A	8 Well-defined 16 Poorly-defined	12 Multiple 12 Single	N/A
Surov et al., 2010 [11]	5 Isointense	5 Hyperintense	5 hypointense	N/A	N/A	5 Homogeneous	N/A	N/A	N/A
Chun et al., 2010 [30]	9 Slightly hyperintense 11 Isointense	N/A	20 Intermediate (isointense)	N/A	N/A	13 Diffuse homogeneous 4 Thick peripheral bandlike 2 Marginal-septal 1 N/A	N/A	14 Multiple 6 Single	15 Yes 5 No
Surov et al., 2014 [35]	8 Isointense	8 Hyperintense	N/A	N/A	N/A	N/A	N/A	N/A	N/A
**Total**	**40 Isointense** **29 Slightly hyperintense** **5 Iso- to slightly hyperintense** **1 Hyperintense** **2 N/A**	**42 Hyperintense** **35 N/A**	**20 Intermediate (Isointense)** **19 Hypointense** **3 Hyperintense** **35 N/A**	**3 Hyperintense** **74 N/A**	**18 Hyperintense** **59 N/A**	**31 Homogeneous** **19 Heterogeneous** **27 N/A**	**22 Poorly-defined** **14 Well-defined** **41 N/A**	**36 Multiple** **25 Single** **16 N/A**	**20 Yes** **5 No** **52 N/A**

^1^ signal intensity compared to muscle tissue; ^2^ signal intensity compared to fat tissue.

## Data Availability

The data attained in this study are available within the article.
